# Enhancing the use of Asthma and COPD Assessment Tools in Balearic Primary Care (ACATIB): a region-wide cluster-controlled implementation trial

**DOI:** 10.1038/npjpcrm.2016.3

**Published:** 2016-03-10

**Authors:** Miguel Román-Rodríguez, Marina Garcia Pardo, Lucia Gorreto López, Ana Uréndez Ruiz, Job FM van Boven

**Affiliations:** 1 IB Salut Balearic Primary Care Health Service, Palma de Mallorca, Spain; 2 Primary Care Chronic Respiratory Research Unit, IdisPa Balearic Health Research Institute, Palma de Mallorca, Spain; 3 Unit of PharmacoEpidemiology & PharmacoEconomics, Department of Pharmacy, University of Groningen, Groningen, The Netherlands

## Abstract

Asthma and chronic obstructive pulmonary disease (COPD) health status assessment tools have demonstrated their value in guiding clinical management. Their use in primary care is still suboptimal. The objective of this study was to assess the effect of an educational intervention programme on the use of the Asthma Control Test (ACT), modified Medical Research Council (mMRC) and COPD Assessment Test (CAT) among primary care settings of the Balearic Islands, Spain. In this region-wide cluster-controlled implementation study, an educational intervention on the use of respiratory health status tools was provided to primary care practices in Mallorca (intervention group). Practices in Ibiza and Menorca functioned as control practices. Written and multimedia materials were provided to all participants to educate their colleagues. Primary outcome was the difference between intervention and control practices in the percentage of practices that increased the use—and recording—of ACT, CAT and mMRC tests between the 6-month period before intervention and the 6-month period after intervention. In the intervention group, 32 out of 45 (71%) centres enhanced the total number of tests, compared with 4 out of 12 (33%) in the non-intervention group (*χ*^2^; *P*=0.02). Before intervention, 399 test scores were recorded in 88,194 patients (asthma: 57,339; COPD: 30,855). After intervention, 1,576 test scores were recorded in 92,714 patients (asthma: 61,841; COPD: 30,873). An educational intervention programme targeted on primary care physicians enhances the use of respiratory health status tools and promotes behavioural changes. However, the effect is very low and difficult to measure in clinical terms.

## Introduction

Recently updated guidelines for asthma and chronic obstructive pulmonary disease (COPD) management recommend the use of health status assessment tools to promote tailored clinical patient management.^1,2^ Health status assessment has even become an integrated part of the classification of patients’ disease severity and serves as a valuable guide for treatment selection.^1,2^ Several control measures and questionnaires have been validated to be used by practitioners and patients to maintain control of asthma and COPD.^3,4^ Among the most widely used COPD health questionnaires in primary care are the modified Medical Research Council (mMRC) dyspnoea scale, COPD Assessment Test (CAT) and the Clinical COPD Questionnaire.^5–7^

In asthma management, the latest Global Initiative for Asthma (GINA) guidelines recommend that treatment should be continuously adjusted, driven by the patient’s asthma control status.^1^ Although several composite control measures exist, one of the most used questionnaires in daily practice is the Asthma Control Test (ACT).^8^

However, in spite of the existence, availability and broad acceptance of these questionnaires and guidelines, there is still evidence of poor familiarity and lack of specific training in assessing patients with asthma and COPD.^9^ Moreover, there is limited literature available that describes the use of questionnaires in real-life primary care practice.^10,11^ To improve respiratory disease outcomes, it is crucial that routine consultations be undertaken by healthcare professionals with appropriate training.^12^ Notably, the British National Review of Asthma Deaths found a patient more likely to have an assessment of control in primary care clinics than if seen in a secondary care clinic with asthma.^13^ Training of healthcare professionals is considered a core component of managing respiratory conditions in primary care.^14^ Therefore, studies aiming to improve the use of health status measurement are in line with the current research needs of the International Primary Care Respiratory Group (IPCRG).^15^

From 2011 onwards, the Balearic Primary Care Health Service provided the possibility to perform and record respiratory health status assessment tests in the electronic primary care patient management system. Subsequently, the Respiratory Diseases Group of the Balearic Society of Primary Care Medicine (Ibamfic) developed a train-the-trainers programme to enhance the use of health status assessment questionnaires to ultimately improve control markers of asthma and COPD.

The aim of this study is to assess the effects of this educational training programme on the use of the ACT, mMRC and CAT among primary care physicians and nurses.

## Results

### Population

**Figure 1:**
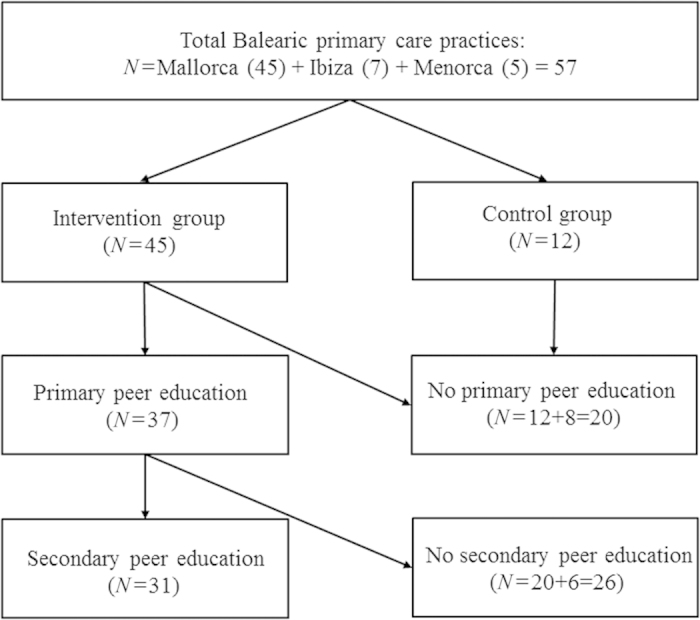
Flow diagram for intervention and non-intervention groups.

In Figure 1, the flow diagram that led to the definition of intervention and non-intervention practices is presented. Comparisons of the baseline characteristics of the two groups are presented in Table 1. Intervention practices had significantly more asthma patients (*P*=0.02), but no difference in the number of COPD patients or health status records was noticed.

### Uptake of the educational programme

Out of the total of 45 primary care settings in Mallorca, 37 participated in the train-the-trainers programme (82%). Of those 37 settings, 31 (84%) performed the secondary peer-to-peer education in their own setting (i.e., six respiratory teams did not provide education to their colleagues).

### Primary outcome: improvement in recording of health status tests at practice level

In the intention-to-educate analysis, in the intervention group, 32 out of 45 (71%) practices enhanced the total number of health status tests performed. In the non-intervention group, 4 out of 12 (33%) practices increased the number of tests performed (*P*=0.02). In the per-protocol analysis, 26 out of all 31 (84%) practices that performed both primary and secondary education enhanced the total number of health status tests performed. In the other group, 10 out of 26 (38%) practices increased the number of tests performed (*P*<0.001).

**Figure 2:**
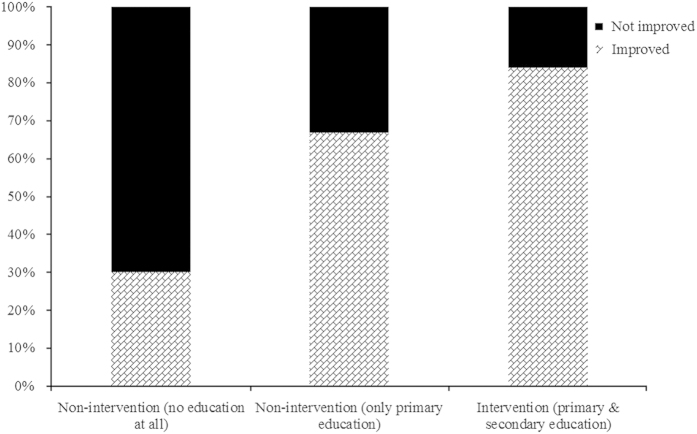
Subgroup analysis of practices, ‘per-protocol’ recruitment (no education at all: *N*=20; only primary education: *N*=6; and both primary and secondary education: *N*=31).

In the six settings that followed the train-the-trainers programme (primary peer-to-peer education), but did not perform the peer-to-peer education (secondary peer-to-peer education), four practices (67%) increased the number of tests recorded (*P*=0.32). Six of the 20 practices that did not participate in the train-the-trainers programme (30%) increased the number of registered tests (*P*<0.001). In the other 14 practices, no change was observed. This is graphically shown in Figure 2.

### Secondary outcome: change in total health status tests recorded

**Figure 3:**
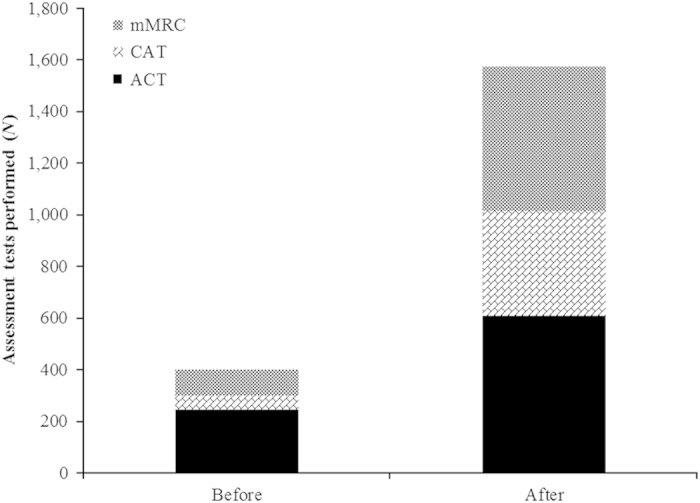
Total asthma and CATs performed before and after intervention (in total Balearic population). ACT, asthma control test; CAT, COPD assessment test; mMRC, modified Medical Research Council.

Before intervention (in 2012), 399 health status scores were recorded in a total of 88,194 patients (asthma: 57,339; COPD: 30,855), implying a percentage of patients with a yearly recorded health status of 0.45%. After intervention (in 2013), 1,576 health status scores were recorded in 92,714 patients (asthma: 61,841; COPD: 30,873), a percentage of 1.70%. This resulted in a significant difference after intervention (*P*<0.0001). An overview is provided in Figure 3.

### Other outcomes

**Figure 4:**
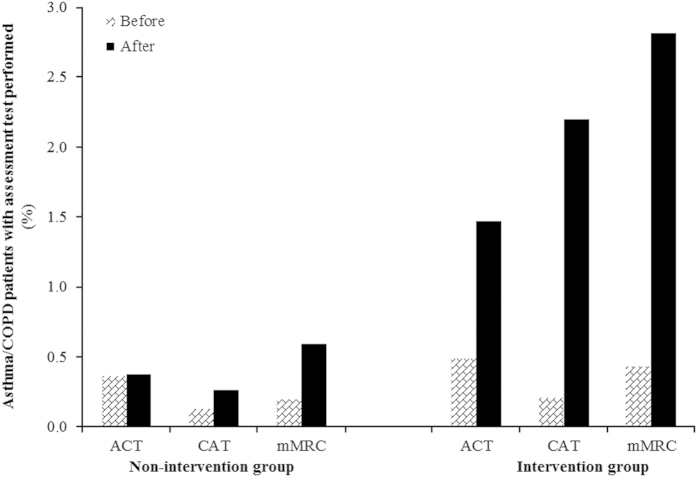
Change in the percentage of asthma/COPD patients with recorded ACT, CAT or mMRC after intervention: intervention practices (*N*=45) versus non-intervention practices (*N*=12) Intention to Educate Analysis. ACT, asthma control test; CAT, COPD assessment test; mMRC, modified Medical Research Council.

In Figure 4, the change in the percentage of asthma (ACT) and COPD (CAT, mMRC) patients with a recorded health status is compared between the intervention and non-intervention groups.

On average, after intervention, there were 20.6 more health status tests recorded per Balearic primary care setting than before intervention. In the intervention practices (*N*=45), an average increase of 24.8 (min: −12; max: +113) tests was noticed (388% increase). In the non-intervention practices (*N*=12), the average increase was 4.9 (min: −12; max: +73; 53% increase). The relative increase in the recording of individual test scores was highest for the mMRC and lowest for the ACT (Figure 4).

## Discussion

### Main findings

Primary care practices that received an educational intervention slightly improved the overall percentage of patients with a record of an ACT, CAT and/or mMRC health status score, compared with practices that did not participate in the educational intervention programme. However, the absolute percentage of patients with an asthma and/or COPD health status score recorded was still relatively low (1.70%), even after intervention.

### Interpretation of findings in relation to previously published work

Few previous studies have been published assessing the effect of implementation of educational programmes targeted on healthcare providers, but the methods and outcomes of these trials are very heterogeneous.^16–23^ Moreover, to our knowledge, studies specifically focusing on the use of respiratory health status assessment tools in primary care are generally lacking. An educational programme in Denmark focusing on improving primary care management of COPD showed that, in line with our study, treatment according to guidelines was suboptimal, but that focused education of general practitioners (GPs) could result in marked improvements in COPD management.^16^ A second study showed that subgroups with lowest starting points showed the highest potential for improvement.^17^ In contrast, a Dutch study that assessed the effect of an educational programme on COPD management, targeted on healthcare providers, showed no effect, which was partly explained by the little room for improvement.^18^ This partly explains our findings, as in our study the overall use of asthma and COPD tools was very low and offered a large initial room for improvement. Also with regard to optimisation of asthma care, GP educational programmes have shown positive effects, but long-term effects, however, could not be established.^19^ In general, recommendations on educational strategies have been established, and they showed that interactive and clinically integrated activities provided the best basis for effective implementation in clinical practice.^20^ In line with this, a review of the literature by Davis *et al.* ^21^ indicated that interactive sessions may indeed change professional behaviour in contrast to solely didactic sessions. In addition, a previous study highlighted the importance of providing protected time for healthcare profesionals to attend educational sessions and to include links to daily clinical practice.^22^ A recent study has shown that multifaceted educational programmes sensitive to local circumstances can reduce antibiotics prescription in primary care.^23^ Our educational training programme used an interactive strategy, was offered to both physicians and nurses during their day-to-day practice, according to local needs, and linked to specific clinical tools and measures.

### Strengths and limitations

Educational strategies are often developed without measuring their real-life impact. Our educational programme included an implementation research process to measure the impact of the intervention before trying to upscale. This study is one of the first studies in this field that attempted to quantitatively show behavioural change after an educational intervention in real-life primary care practice by assessing the use of respiratory health status tools. Yet, cutoffs for minimal clinically relevant improvements need to be established, to promote meaningful interpretation. A major strength of our study was the real-life setting, covering the complete regional population, in which this study was conducted. The design, including clustering by islands, reduces contamination between the groups. The same electronic system is used in all primary care settings by both doctors and nurses, assuring no missing data on questionnaires performed and registered. As a result of this setting, our study not only provides insight into the potential effectiveness of the educational programme, but it also provides estimates of expected uptake among healthcare providers. In addition, it is important to publish numbers and percentages of the current use of asthma and COPD assessment tools in daily primary care practice. The educational intervention included strategies to maximise the number of attending healthcare providers: timing of the education (i.e., it was offered during regular working hours), a financial incentive, peer-to-peer education developed by primary care doctors and nurses (familiar faces and clinical problems), and easy-to-reach locations, situated near the majority of primary care practices.

Despite these strengths, the setting and the non-randomised design of this study have their limitations. On the one hand, the real-life setting represented daily practice behaviour and effectiveness, and on the other hand one can argue that this influenced the strength of the evidence for efficacy of the education programme. Different asthma and COPD prevalence rates among practices may have biased the results. The relatively higher number of asthma patients may have led to more experience with the ACT in the intervention group. The Hawthorn effect could have had a role, but the difference between intention-to-educate and per-protocol analyses seems to suggest that this was only minor. Furthermore, apart from the relatively scarce use of the health status tools, some of them might not have been registered in the electronic files. Last, the 6 months' time period may be considered relatively short to assess whether the effects were sustained over time.

### Implications for future research, policy and practice

The educational programme described in this study is considered generalisable and feasible to implement in other primary care settings, as the system of specialised GPs and primary care nurses is not unique to Spain but is also applied in, for example, The Netherlands and the United Kingdom. A previous educational intervention in asthma showed that translation to other countries was successful;^18^ however, more studies on the transferability of successful interventions to other regions, cultures or systems are recommended.^24^ Despite the small clinical impact of the intervention, the fear of low clinical impact of an educational programme should not be a reason for not measuring any clinical outcome.

To complement continuity of care, asthma and COPD health status tools could be used as an integrated part of daily practice and each GP’s regular patient assessment. Therefore, more studies on the long-term effects of these types of educational interventions are needed. For optimal effectiveness, we recommend to reinforce the education periodically and to provide GPs with feedback on their own results.^25^ As previously shown, for continuous implementation, sufficient time and financial resources need to be available.^26^ The Quality Outcomes Framework (http://www.hscic.gov.uk/qof), an annual reward and incentive programme detailing GP practice achievement results, was introduced in 2004 in the UK and has obtained positive results regarding primary care professionals’ behavioural changes and clinical outcomes. However, in the Balearic Islands and other regions heavily affected by the economic crisis, it is difficult to get financial incentives from the national health systems, and therefore these kinds of educational programmes could be considered. Before upscaling these kinds of educational programmes, further research on factors and incentives to participate is needed.^25^ Last, it is important to take into account the economic impact of these types of educational interventions, as the financial burden of asthma and COPD is considerable and therefore treatment budgets should be spent wisely.^27,28^ As such, with regard to the cost-effectiveness of these programmes, we expect that targeting on populations most in need (i.e., with the largest room for improvement) offers the highest potential for positive and cost-effective results.^17^

### Conclusions

An educational intervention programme targeted on primary care physicians enhances the use of respiratory health status tools and promotes behavioural changes. However, the effect is very low and difficult to measure in clinical terms.

## Materials and methods

### Study design

This is a region-wide, cluster-controlled implementation trial. The Balearics comprise three main islands with 57 primary care practices, which are similar in healthcare system. The clusters were defined geographically (by island). All practices in Mallorca (*N*=45) were offered an educational training and implementation programme on the use of respiratory health status tools. All practices in Ibiza and Menorca (*N*=12) were not offered any training programme.

To assess the effect of the intervention, two cross-sectional assessments on the use of respiratory health status tools were performed. The first was performed exactly 6 months before the educational intervention. To be consistent, we performed the second assessment over the 6-month period after the educational intervention, so that the total study period was 1 year (November 2012–2013). No other similar educational activities were developed during the 6 months before or after intervention, and there was no incentive or communication at all that promoted the recording of health status tools. In the cross-sectional assessments, intervention practices (*N*=45) and non-intervention practices (*N*=12) were compared.

### Setting and population

In the Balearics, every setting has a ‘respiratory team’, consisting of a GP and a nurse. Every year this team receives a 5- h accredited workshop in respiratory care. It is expected that they provide respiratory training to their fellow practice colleagues (up to ~10 GPs and nurses per primary care setting). These teams were invited by the Balearic Health Service training Unit to participate in a train-the-trainers programme on the use of respiratory questionnaires.

### Data source

Data from all Balearic practices (Mallorca, Ibiza, Menorca) were centrally extracted from the electronic primary care patient management system (e-SIAP)^29^ and anonymised. The e-SIAP system is used by all primary care settings in the Balearics. It includes information such as diagnoses by ICD-9 codings, clinical parameters and information about the performance, dates and scores of the asthma, and COPD questionnaires.

### Sample size

A sample size calculation was not necessary, as data of the complete Balearic population (all 57 practices) were obtained by direct extraction from the primary care system.^29^

### Questionnaires

Since 2011, the three health status assessment questionnaires ACT, CAT and mMRC are available in e-SIAP to be used by any clinician working in the Balearic public primary care setting. A short description of each questionnaire is provided below.

#### ACT

The ACT is a 5-item questionnaire that can be filled out by asthma patients either online, by telephone or on paper. It covers questions on functional impairment, shortness of breath, night-time symptoms, use of rescue medication and self-perceived asthma control. All items are scored on a 1–5 scale, resulting in a total score of asthma control that ranges between 5 and 25. A scoring below 20 indicates non-controlled asthma.^30^ The ACT has been extensively validated, it is translated in Spanish and a minimal important difference has been established (two for children, three for adults).^4,31,32^

#### CAT

The CAT provides a measure of the impact of COPD on patient’s health status.^6^ It is a validated 8-item questionnaire with scores on each question ranging between 0 and 5. Summing all item scores results in a total score of between 0 (no impact) and 40 (very high impact). The minimal clinically important difference of the CAT score has been suggested to be ~2 units,^33^ and it has good properties to be used in primary care.^34^

#### mMRC

Measuring the level of dyspnoea is essential to assess severity and to assist in the selection of pharmacologic treatment for patients with COPD. To quantify the impact of breathlessness, the Medical Research Council dyspnoea scale (MRC) has been developed.^5^ A slightly modified version (mMRC) is now widely used in current clinical practice, mainly to define functional status.^10^ This 1-item questionnaire defines 5 levels of dyspnoea severity, ranging from 0 (breathless with strenuous exercise) to 4 (too breathless to leave the house or when dressing). It is one of the severity markers used to define GOLD status and BODE and DOSE prognosis indexes.

### Educational programme

#### Strategy

This educational intervention consisted of a train-the-trainers programme that took place in April 2013, during regular working hours. In this programme, the local respiratory team at each primary care setting was trained to educate their fellow practice colleagues on the use of asthma and COPD assessment tools.

#### Train-the-trainers programme

The train-the-trainers programme took place in April 2013, during regular working hours. In this programme, the ‘respiratory team’ of each primary care setting received training from the Ibamfic's respiratory expert in the use of the ACT, the CAT and the mMRC questionnaires (primary peer-to-peer education). The interventions were half day, and they were developed by a team (doctor and nurse) for a group of no more of 20 attendees. The training was divided into a clinical session and a technical session. First, using the format of an interactive clinical session, the importance of the use of clinical health status questionnaires was explained (assessment of disease control, measuring the degree of disease severity, their help in guiding and selecting treatment and the usefulness of the information regarding their patient’s benefits). To illustrate their usefulness, several real-life daily clinical cases from their peer-colleagues were presented and discussed. Subsequently, each questionnaire was discussed in detail (e.g., by explaining the meaning of each question and the scoring system). In the second part of the workshop, technical information was provided on where to find the questionnaires in the patient management system and how to record the scores.

Finally, at the end of the primary educational session, written and multimedia material was provided to all participants, and they were encouraged to organise a session to educate their fellow practice colleagues (secondary peer-to-peer education). The secondary education took place in their own primary care centres during the day-to-day continued educational activities and consisted of the same elements as the primary intervention. A financial incentive of 50 euros was offered to each team if they performed this session within 2 months after completing the workshop, and they were reminded to perform it during this period. The primary care teams were informed that their recording of the questionnaires would be tracked and assessed in the current study. No additional information was given to the non-participating practices.

### Participating practices

All the respiratory teams from the 57 primary care practices in the Balearics (including over 400 different GPs and nurses) participated in the study. The intervention practices in Mallorca (45) were invited for the train-the-trainers programme, whereas the practices in Menorca and Ibiza (12) were not invited because of geographical reasons and therefore functioned as natural non-intervention practices.

To check whether there were significant baseline differences between groups regarding patient population size and the previous use of the health status tools, we compared these baseline characteristics between the intervention and the non-intervention groups.

Results from all Balearic primary care practices (whether or not participating in the training programme) were included in the two cross-sectional assessments on the use of respiratory health status questionnaires, thereby providing a real-life, region-wide and representative measure.

### Outcomes

Primary outcome was the difference between intervention and non-intervention practices in the percentage of practices that increased the use—and recording—of ACT, CAT and mMRC tests between November 2012 (before intervention) and 2013 (after intervention) using an intention-to-educate analysis. In addition, a per-protocol analysis was performed, which was a sub-analysis of the primary outcome; we defined three groups: practices completing both the primary and secondary educational programme, practices that only completed the primary education and non-intervention practices.

The secondary outcome was the total number of tests recorded before and after intervention, as the percentage of the total number of asthma and COPD patients in all the Balearic practices.

Other outcomes included the difference in average change in number and percentage of patients (from total asthma or COPD population) with a recorded ACT, CAT or mMRC between the intervention and the non-intervention group.

### Statistics

Student’s *t*-tests were used to compare continuous variables (e.g., baseline characteristics). *Χ* ^2^ tests were used to assess the difference between intervention and non-intervention practices and also for the other comparisons (e.g., between practices with primary education only and those with primary and secondary education). A *P* value<0.05 was considered significant.

## Figures and Tables

**Table 1:** Baseline characteristics of the intervention group and the non-intervention group

	*Intervention group (*N*=45)*	*Non-intervention group (*N*=12)*	P *value*^a^
Mean number of asthma patients per practice (s.d.)	1,071 (411)	762 (335)	0.02
Mean number of COPD patients per practice (s.d.)	562 (212)	464 (185)	0.15
Mean number of ACT, CAT and MRCs registered per practice (mean, s.d.)	6.4 (15.3)	9.3 (12.3)	0.55

Abbreviations: ACT, asthma control test; CAT, COPD assessment test; COPD, chronic obstructive pulmonary disease; MRC, Medical Research Council.

a Two tailed unpaired *t*-test.
